# Approximation to the economic cost of healthcare for hypertensive patients diagnosed with COVID-19

**DOI:** 10.3389/fpubh.2024.1333081

**Published:** 2024-03-18

**Authors:** Jesús Calderón-Moreno, María Jesús Delgado-Rodriguez, Raúl Juárez-Vela, Clara Isabel Tejada-Garrido, Regina Ruiz de Viñaspre-Hernández, Amaya Burgos-Esteban, Pilar Sánchez-Conde, Vicente Andreu-Fernández, Vicente Gea-Caballero, Jose Angel Santos-Sanchez, Manuel Quintana-Diaz, Eva María Andrés-Esteban

**Affiliations:** ^1^Business Economics Department, University of Rey Juan Carlos, Madrid, Spain; ^2^Department of Nursing, GRUPAC, University of La Rioja, Logroño, Spain; ^3^Research Group Blood Patient Management, IDI-Paz Research Institute, Madrid, Spain; ^4^Faculty of Medicine, University of Salamanca, Salamanca, Spain; ^5^Anesthesia and Resuscitation Service, University Healthcare Complex, Salamanca, Spain; ^6^Faculty of Health Sciences, International University of Valencia, Valencia, Spain; ^7^Intensive Care Unit, Hospital La Paz, La Paz, Spain

**Keywords:** healthcare economics and organizations, economics, hospital, COVID-19, public health administration, cost of illness

## Abstract

**Introduction:**

Many researchers have focused their studies on hypertension due to its over-representation among COVID-19 patients. Both retrospective and observational studies conducted close to the Wuhan area have reported that hypertension is the most common comorbidity observed in patients affected by COVID-19.

**Objective:**

Our objective is that patients with arterial hypertension have a worse prognosis in terms of evolution leading to higher costs.

**Methods:**

A retrospective cross-sectional study was conducted. A total of 3,581 patients from La Paz University Hospital (LPUH) during the period between 15 July 2020 and 31 July 2020 were included in this study.

**Results:**

It should be noted that 40.71% of the patients were hypertensive. As expected, hypertension was associated with men, among whom we observed a higher prevalence and a higher age (median age of 77 years (IQI: 65–85) versus 52 years (IQI: 37–64), *p*-value < 0.001). Hypertensive patients had a higher prevalence of dyspnea (52.14% vs. 47.15%, *p*-value = 0.004) and altered awareness (14.89% vs. 4.30%, *p*-value <0.001). The non-parametric Kaplan–Meier curve estimates the survival of patients in the two study groups. We can see how patients with hypertension have a higher associated mortality, with the difference being statistically significant, *p*-value (log-rank) = 0.004. Only for the appearance of complications during hospitalization, the group of hypertensive patients reached the figure of €1,355,901.71 compared to the total of 421,403.48 € for normotensive patients.

**Conclusion:**

Our study shows the worse clinical evolution of patients with COVID-19 in terms of associated morbidity and mortality. It also shows that the cost of managing patients with hypertension is greater than that of managing normotensive patients.

## Introduction

Many researchers have focused their studies on hypertension due to its over-representation among COVID-19 patients (1). Both retrospective and observational studies conducted close to the Wuhan area have reported that hypertension is the most common comorbidity observed in patients affected by COVID-19, ranging between 15 and 30% (2–4). In one of the largest studies conducted in Wuhan with data collected from 1,099 COVID-19 patients, 165 patients (approximately 15% of the total sample) had high blood pressure (5). The same study also reported that a total of 23.7% of hypertensive patients had higher disease severity than 13.4% of normotensive subjects. However, 35.8% of hypertensive patients experienced worst outcomes in terms of intensive care unit (ICU) admission, mechanical ventilation, or death compared to just 13.7% of normotensive patients (5).

Another study conducted in China, which investigated 138 COVID-19 patients, found a similarly high prevalence of hypertension among the patients (31.2%) (2). The researchers also affirmed that 58.3% of hypertensive patients with COVID-19 infection were admitted to ICU compared to 21.6% of patients with normal blood pressure. Guan et al. studied a cohort of 1,590 patients from 575 hospitals and found that hypertension was independently associated with severe COVID-19 (hazard ratio 1.575; 95% CI: 1.07–2.32). All these findings indicate that hypertensive patients have a higher risk of developing severe outcomes from COVID-19.

These early results were similar to those subsequently found in other countries. Thus, JAMA published data on 1,591 patients admitted to intensive care units in Italy (6). Arterial hypertension (49%) and cardiovascular disease (21%) were the most frequent comorbidities, above other respiratory diseases. The study stratified the cohort by the presence or absence of hypertension and hypertensives finding that patients with arterial hypertension had higher mortality (65% vs. 40%, *p* < 0.001).

The Spanish National Health System (SNS) is based on a Beveridge-type public model (7). It is a decentralized national health system, with competencies transferred to 17 Spanish autonomous communities (regions), under the control of the Ministry of Health. The SNS coverage gradually spread until 100% of citizens were covered in 1989. Currently, care is financed by taxes, and services are accessed by health cards. There are not many studies related to the cost of managing patients infected with the SARS-CoV-2 virus. In Spain, Calderon et al. reported that the cost of managing patients with COVID-19 without hospitalization is €729.79, and the cost of hospitalized patients ranges between €4294.36 and €14440.68, if there is an ICU admission (8).

If we focus on the worse evolution of hypertensive patients, they have a higher incidence of complications, longer hospital stays, or stays in intensive care units than normotensive patients, and all this translates into higher healthcare costs for the management of hypertensive patients when infected with the SARS-CoV-2 virus. Therefore, our working hypothesis is that patients with arterial hypertension have a worse prognosis in terms of evolution leading to higher costs.

## Methods

### Design

A retrospective cross-sectional study was carried out.

### Data collection

Given the avalanche of patients in the emergency department, wards, and the ICU and the lack of knowledge of the disease during the initial months of the pandemic, the Hospital Universitario La Paz created a specific data collection notebook for all those who came to the hospital with clinical manifestations compatible with SARS-CoV-2 virus infection. This database was created by several experts in epidemiology at the hospital with the aim of learning more about the disease known as COVID-19.

### Population and sample

A total of 3,581 patients from the La Paz University Hospital (LPUH) during the period between 15 July 2020 and 31 July 2020 were included in this study. The database included sociodemographic data, clinical status, laboratory findings, and clinical management of patients admitted with a respiratory infection caused by SARS-CoV-2 since the outbreak of the current pandemic.

### Variables

Patient demographic data were collected prior to admission. Total costs were categorized based on care settings (admission to the hospital ward, admission to the ICU, and length of hospital stay) and the occurrence of most frequent complications (such as respiratory infection, pneumonia, acute respiratory distress syndrome, pneumothorax, pleural effusion, meningitis, convulsions, stroke, heart failure, endocarditis, arrhythmia, cardiac ischemia, cardiac arrest, coagulation problems, anemia, renal failure, pancreatitis, hepatic failure, psychiatric illness, and gastrointestinal bleeding). Additionally, variables associated with chronic health problems (such as smoking, diabetes, hypertension, chronic cardiac diseases, asthma, and chronic bronchitis) were considered. These variables have been linked to complications in COVID-19.

The cost data used were provided by the accounting department of the hospital, which allowed more precise estimates to be made.

### Statistical analysis

Quantitative variables were described using robust statistics, such as median and interquartile interval, whereas for qualitative variables, frequency distribution was used. For the comparison of quantitative variables that were not normally distributed among frailty groups, the Kruskal–Wallis non-parametric H test was used, based on the Shapiro–Wilk test. Finally, the chi-squared test was used to compare qualitative variables.

The survival estimate was assessed using the Kaplan–Meier method comparing the survival curve between the groups with the log-rank test. The multivariate analysis was carried out by means of Cox regression, with the forward conditional method, introducing as independent variables the variables that obtained statistical significance in the bivariate analysis or that could have a clinically plausible implication. The results of the multivariate model were presented as a hazard ratio (95% CI).

The statistical analysis was performed using STATA v16.0, and a *p*-value of 5% was considered statistically significant.

### Ethical considerations

The study was conducted in accordance with the principles outlined in the Declaration of Helsinki (2008 update, available on the World Medical Association website).^1^ Additionally, it adhered to the standards of good clinical practice as described in the ICH Harmonized Tripartite Guidelines for Good Clinical Practice (2001) and the Guidelines for Good Epidemiological Practice.^2^ The study was approved by the Clinical Research Ethics Committee of LPUH, Madrid, with the LPUH code: PI-4155. It was not necessary to provide a formulary of informed consent as the anonymized database was used for data extraction. This study was conducted in accordance with European and Spanish regulations for the protection of personal data (Organic Law 3/2008).

## Results

### Description of the sample

The patients were stratified according to the diagnosis of hypertension. Table 1 shows the demographic characteristics of these patients. It should be noted that 40.71% of the patients were hypertensive. As expected, hypertension was associated with men, among whom we observed a higher prevalence and a higher median age [median of 77 years (IQI: 65–85) vs. 52 years (IQI: 37–64), *p*-value <0.001].

**Table 1:** Description of demographic variables in patients with and without arterial hypertension admitted for COVID-19.

Variable	Hypertension		*p*-value
	No	Yes	
N	2,123 (59.29%)	1,458 (40.71%)	
Sex			< 0.001
Male	926 (44.80%)	799 (54.80%)	
Female	1,141 (55.20%)	659 (45.20%)	
Age (median, IQI)	52 (37–64)	77 (65–85)	< 0.001
Healthcare worker	601 (30.23%)	67 (4.83%)	< 0.001
Type overcrowding			< 0.001
Without overcrowding	1941 (94.68%)	1,222 (84.80%)	
Residence	100 (4.88%)	214 (14.85%)	
Hotel	8 (0.39%)	5 (0.35%)	
Prison	1 (0.05%)	0 (0.00%)	
Relationship with positive COVID	333 (17.48%)	221 (16.54%)	0.485
Suspected nosocomial transmission	702 (34.50%)	362 (25.02%)	< 0.001
Functional stage			< 0.001
Dependent for basic activities of daily living	107 (5.35%)	145 (10.48%)	
Semi-dependent for basic activities of daily living	56 (2.80%)	134 (9.68%)	
Independent for basic activities of daily living	1838 (91.85%)	1,105 (79.84%)	

The clinical presentation of COVID-19 symptomatology was also very different between patients with hypertension and normotensive patients, as shown in the following Table 2. Notably, hypertensive patients exhibited a higher prevalence of dyspnea (52.14% vs. 47.15%, *p*-value = 0.004) and altered awareness (14.89% vs. 4.30%, *p*-value <0.001).

**Table 2:** Clinical symptomatology at hospital admission.

Symptoms	Hypertension		*p*-value
	No	Yes	
Fever	1,435 (71.68%)	999 (68.90%)	0.077
Headache	525 (26.25%)	141 (9.74%)	< 0.001
General discomfort	819 (40.91%)	603 (41.61%)	0.678
Myalgias	645 (32.30%)	273 (18.85%)	< 0.001
Rhinorrhea	136 (6.81%)	57 (3.94%)	< 0.001
Dysgeusia	345 (17.36%)	102 (7.05%)	< 0.001
Anosmia	355 (17.78%)	87 (6.02%)	< 0.001
Cough	1,275 (63.59%)	814 (56.14%)	< 0.001
Productive cough	258 (12.97%)	235 (16.36%)	0.005
Odynophagia	240 (12.02%)	67 (4.64%)	< 0.001
Thoracic pain	225 (11.26%)	114 (7.87%)	0.001
Chest pain	62 (3.10%)	32 (2.21%)	0.112
Hemoptysis expectoration	23 (1.15%)	17 (1.17%)	0.946
Dyspnea	944 (47.15%)	756 (52.14%)	0.004
Abdominal pain	106 (5.30%)	63 (4.35%)	0.204
Diarrhea	449 (22.43%)	319 (22.00%)	0.766
Nausea	187 (9.35%)	130 (8.98%)	0.714
Vomiting	162 (8.09%)	100 (6.90%)	0.191
Alteration of level awareness	86 (4.30%)	216 (14.89%)	< 0.001
Alteration of level behavior	22 (1.10%)	29 (2.00%)	0.030
Convulsion	2 (0.10%)	3 (0.31%)	0.415

Regarding the complications presented by patients with hypertension, we observed a significant difference in most of them. Acuate Respiratory Syndrome by COVID-19, much more present in patients with HT (31.37% vs. 12.00%, *p*-value <0.001), pneumothorax (6.73% vs. 0.67%, *p*-value = 0.007), cardiac arrest (15.38% vs. 0.00%, *p*-value <0.001), acute confusional syndrome (29.81% vs. 4.67%, *p*-value <0.001), and clotting alteration or sharp failure as shown in Table 3. In addition, hypertensive patients have a higher in-hospital mortality rate than normotensive patients (Tables 4, 5).

**Table 3:** Incidence of complications during hospital stay.

Complications	Hypertension		*p*-value
	No	Yes	
Infections	25 (16.00%)	34 (32.69%)	0.002
Infections with microorganisms determined	14 (9.33%)	25 (24.04%)	0.001
Bacterial pneumonia	6 (4.00%)	13 (12.50%)	0.011
Acute respiratory syndrome	18 (12.00%)	33 (31.73%)	<0.001
Pneumothorax	1 (0.67%)	7 (6.73%)	0.007
Effusion pleural	2 (1.33%)	4 (3.85%)	0.195
Meningitis	0 (0.00%)	3 (2.88%)	0.036
Convulsions	0 (0.00%)	1 (0.96%)	0.229
Stroke	1 (0.67%)	2 (1.92%)	0.362
Congestive cardiac inflation	3 (2.00%)	4 (3.85%)	0.377
Myocarditis	0 (0.00%)	1 (0.96%)	0.229
Pericarditis	1 (0.67%)	0 (0.00%)	0.404
Endocarditis	0 (0.00%)	0 (0.00%)	NA
Arrhythmia	2 (1.33%)	8 (7.69%)	0.010
Cardiac ischemia	1 (0.67%)	1 (0.96%)	0.794
Cardiac arrest	0 (0.00%)	16 (15.38%)	< 0.001
Bacteremia	7 (4.67%)	13 (12.50%)	0.023
Clotting alteration	5 (3.33%)	20 (19.23%)	< 0.001
Anemia subsidiary	4 (2.67%)	6 (5.77%)	0.211
Rhabdomyolysis	2 (1.33%)	3 (2.88%)	0.381
Sharp failure	8 (5.33%)	38 (36.54%)	< 0.001
Pancreatitis	1 (0.67%)	0 (0.00%)	0.404
Hepatic failure	2 (1.33%)	6 (5.77%)	0.047
Acute confusional syndrome	7 (4.67%)	31 (29.81%)	< 0.001
Psychiatric complications	4 (2.67%)	4 (3.85%)	0.597
Adverse reaction drugs	13 (8.67%)	8 (7.69%)	0.782
Severe adverse drug reaction	1 (0.67%)	3 (2.88%)	0.163
Exitus	204 (10.52%)	438 (30.54%)	< 0.001
Hospital stays	11 (9–19)	10 (7–14)	0.296

*Median and interquartile range.

**Figure 1:**
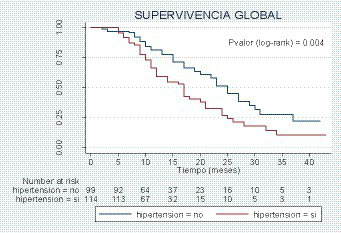
Kaplan–Meier estimator.

Figure 1 shows the non-parametric Kaplan–Meier curve, which estimates the survival of patients in the two study groups. We can see how patients with hypertension have a higher associated mortality, with the difference being statistically significant, *p*-value (log-rank) = 0.004.

**Table 4:** Cox regression model to predict COVID-19 mortality.

			CI (95%)	
	HR	*p*-value	Lower	Superior
Chronic heart disease	1.70	0.030	1.05	2.75
Chronic renal disease	2.08	0.018	1.13	3.80
Hypertension	1.67	0.024	1.07	2.62

C – Harrell = 0.632. HR, hazard ratio; CI, confidence interval.

**Table 5:** Costs associated with the management of COVID-19 patients with and without hypertension.

Table of costs per person	Hypertension	
	No	Yes
Infections	65.070,25 €	88.495,54 €
Infections with microorganisms determined	853,86 €	1.524,75 €
Bacterial pneumonia	22.218,00 €	48.139,00 €
MRS	60.557,22 €	111.021,57 €
Pneumothorax	2.649,75 €	18.548,25 €
Effusion pleural	8.108,06 €	16.216,12 €
Meningitis	- €	6.684,00 €
Convulsions	- €	3.057,00 €
Stroke	3.572,34 €	7.144,68 €
Congestive cardiac inflation	8.670,00 €	11.560,00 €
Myocarditis	- €	3.214,00 €
Pericarditis	2.079,00 €	- €
Endocarditis	- €	- €
Arrhythmia	4.906,00 €	19.624,00 €
Cardiac ischemia	2.430,00 €	2.430,00 €
Cardiac arrest	- €	51.040,00 €
Bacteremia	19.299,00 €	35.841,00 €
Coagulation alteration	20.045,00 €	80.180,00 €
Anemia	15.852,00 €	23.778,00 €
Rhabdomyolysis	23.264,00 €	34.896,00 €
Sharp failure	25.384,00 €	120.574,00 €
Pancreatitis	9.807,00 €	- €
Hepatic failure	6.170,00 €	18.510,00 €
Acute confusional syndrome	39.627,00 €	175.491,00 €
Psychiatric compliances	29.536,00 €	29.536,00 €
Adverse reaction drugs	45.500,00 €	28.000,00 €
Severed adverse drug reaction	5.805,00 €	17.415,00 €
Hypertension		402.981,80 €
TOTAL COMPLICATIONS	421.403,48 €	1.355.901,71 €
Cost of hospital stay	7.491,00 €	6.810,00 €
Total patient management	428.894,48 €	1.362.711,71 €
Total handling per person	202,02 €	934,64 €

In the Cox regression model, we observed that chronic cardiac disease (HR = 1.70, 95% CI: 1.05–2.75), chronic kidney disease (HR = 2.08, 95% CI: 1.13–3.80), and arterial hypertension (HR = 1.67, 95% CI: 1.07–2.35) were associated with in-hospital mortality.

Finally, to conclude the analysis, we include the cost of managing complications. We have already seen that hypertensive patients have more complications compared to normotensive patients, and this increases the healthcare costs of these patients. Specifically, only for the appearance of complications during hospitalization, the group of hypertensive patients reached the figure of €1,355,901.71 compared to the total of €421,403.48 for normotensive patients. This difference in figures means that the cost of managing the complications observed in hypertensive patients is 3.2 times higher than in normotensive patients. However, there was no significant difference in hospital stay between the two groups.

## Discussion

Our data show that the group of hypertensive patients studied are older and more frequently men. These two characteristics, male sex and advanced age, are considered as highly relevant conditions associated with a worse prognosis in the evolution of COVID-19. Furthermore, older age is related to higher incidence of comorbidities other than hypertension, such as diabetes, cardiovascular disease, cerebrovascular disease, or obesity, which also increase susceptibility to infection and worsen disease progression. Consequently, the impact of hypertension on COVID-19 is not well defined. However, findings of some studies suggest that hypertension alone may not increase the risk of infection and complications of COVID-19 including death (9).

Many studies show that the cost of managing hypertensive patients is double that of normotensive patients. Our studies are in line with the findings of Badia et al. (10) and it is important to establish an evaluation of interventions and services aimed at the management of hypertension with special emphasis on complications. Numerous studies, such as the analyses presented in *The Lancet* (11), focus on improvements in detection and treatment with the aim, precisely, of reducing prevalence through early diagnosis, thereby reducing complications, which our study has quantified. For example, obesity is an important aspect to take into account in the development of arterial hypertension. Different studies have quantified that, if managed at an aggregate level, the savings in healthcare costs would amount to between €1,859 and €1,926 per person, and the return on investment would be between 3.3 and 7.0%. (12)

However, if we take a global view of hypertension, we know precisely that several studies (13) reflect the comorbidity of patients with hypertension together with other pathologies, such as dyslipidemia, diabetes, ischemic heart disease, and even stroke, which is also reflected in our study when discussing associated comorbidities. Precisely in the field of stroke, hypertension is the main driver of cerebral small vessel disease (CSVD) leading to cognitive impairment and lacunar stroke (14). On the other hand, the COVID-19 pandemic has also highlighted the worse evolution of patients with COVID-19 and the associated costs (8). Precisely, the association between cardiovascular pathology and poor evolution of SARS-CoV-2 infection is striking in terms of costs. With respect to comorbidities and COVID-19 and its associated costs, studies published in different countries (14) show that hypertension, diabetes, cerebrovascular disease, and ischemic heart disease are markedly more frequent in patients who require critical care or die from COVID-19, establishing a causal link between an underlying pathology, such as hypertension, and other factors, such as myocardial dysfunction produced by SARS-CoV-2.

Continuing with myocardial dysfunction, special interest should alert us to the relationship between hypertension and heart failure. It is precisely in the development of left ventricular hypertrophy and consequently heart failure (15) that hypertension is a key factor, and early diagnosis and treatment are necessary. Hypertensive heart disease describes a spectrum of diseases ranging from uncontrolled hypertension to the final development of heart failure, being mainly among others, the triggering event of left ventricular hypertrophy a hypertensive heart disease, something that can be reversible if recognized early and treated aggressively. Regarding heart failure, a study in the United States (16) found that the mean total cost ± SD was $13,807 ± 24,145, with mean total costs of $15,618 ± 25,264 for patients with 30-day readmission and $11,845 ± 22,710 for patients without readmission. These findings are consistent with our study, which also found both the higher costs associated with hypertension and readmission.

Another aspect to be addressed is the complications that end in fatal consequences. We have already mentioned the relationship between hypertension and stroke, stroke being a major public health problem and a problem that accounts for 10% of all deaths (17) Different studies (18, 19) established that approximately half of the patients analyzed had hypertension.

There are many factors to take into account when discussing hypertension and its associated costs. It has been well established that patients with hypertension have greater associated comorbidities, are admitted to hospital more often, and have higher morbidity and mortality rates.

## Limitations

This study has limitations: first, inherent to the type of study, it cannot evaluate cause and effect relationships. Another limitation is that the study was a single-center study mainly due to the imminent need to know the evolution of the disease, and the costs in different autonomous communities or regions may vary. However, as they are public hospitals, the prices are usually subject to the Official State Gazette; therefore, we state that these variations are slight.

Finally, the creation of a specific The Electronic Data Collection Notebook (CRD) for the hospital itself and for this disease in particular means that we cannot compare it with other types of infections such as Influenza.

## Conclusion

The results obtained provide evidence that tends to support our initial hypothesis that hypertensive patients admitted for SARS-CoV-2 respiratory infection would have a worse prognosis in terms of outcomes compared to normotensive patients, leading to higher healthcare costs for the hypertensive patient group. Our study shows the worse clinical evolution of patients with COVID-19 in terms of associated morbidity and mortality. It also shows that the cost of managing the complications observed in hypertensive patients is 3.2 times higher than in normotensive patients.

## Data availability statement

The raw data supporting the conclusions of this article will be made available by the authors, without undue reservation.

## Ethics statement

The studies involving humans were approved by the Clinical Research Ethics Commitee of LPUH, Madrid, with LPUH code: PI-4155. The studies were conducted in accordance with the local legislation and institutional requirements. Written informed consent for participation was not required from the participants or the participants’ legal guardians/next of kin in accordance with the national legislation and institutional requirements.

## Author contributions

JC-M: Conceptualization, Data curation, Formal analysis, Investigation, Methodology, Project administration, Software, Validation, Writing – original draft, Writing – review & editing. MD-R: Investigation, Project administration, Resources, Validation, Visualization, Writing – original draft, Writing – review & editing. RJ-V: Funding acquisition, Methodology, Project administration, Resources, Visualization, Writing – original draft, Writing – review & editing. CT-G: Validation, Visualization, Writing – original draft. RV-H: Funding acquisition, Methodology, Writing – original draft, Writing – review & editing. AB-E: Funding acquisition, Project administration, Writing – original draft. PS-C: Investigation, Supervision, Validation, Writing – original draft. VA-F: Data curation, Investigation, Validation, Writing – original draft. VG-C: Data curation, Writing – original draft. JS-S: Writing – original draft, Investigation. MQ-D: Project administration, Supervision, Writing – original draft, Writing – review & editing. EA-E: Data curation, Formal analysis, Investigation, Methodology, Project administration, Software, Supervision, Validation, Writing – original draft, Writing – review & editing.

## Footnotes

^1^ http://www.wma.net/e/policy/b3.htm

^2^ http://www.ieaweb.org/GEP07.html
